# New Incision for Exposure of Lower Abdomen and Pelvis

**Published:** 1918-12

**Authors:** 


**A New Incision for Exposure of the Lower Abdomen and Pelvis.** By JOHN W. CHURCHMAN, M. D. *Annals of Surgery*, February, 1918.

The lateral oblique incision here described is regarded by the author as an incision of choice whenever wide exposure of the lower abdomen and pelvis is required, as in war wounds of the bladder. It may be uni- or bilateral according to the necessities of the operation; when it is bilateral, the edges of the incision fall apart, and little retraction is needed for the roomiest possible exposure.

(1) *Skin Incision* begins at the symphysis pubis, runs in the mid-line upwards for two inches or more, then diagonally upward and outward, toward the anterior superior spine.

(2) *Fascial Incision* repeats the direction of the skin incision, dividing the rectus sheath longitudinally, then crossing obliquely the anterior sheath and dividing the fascia of m. obliquus externus exactly in the direction of its fibers and as high toward the anterior superior spine as desired. The dividing of the anterior sheath of the rectus must be performed right down to the bone; at the symphysis pubis in operating on the bladder.

(3) *Muscle Incision.* The rectus muscle is freed and divided between clamps. The epigastric vessels are clamped, divided and ligated separately.

(4) The fascia of the m. transversalis and m. obliquus internus and the peritoneum are divided by an incision which repeats the direction of the skin incision.

*Closure.* (a) The peritoneum is sutured by a running stitch.

(b) The divided rectus muscle may be approximated by mattress sutures. A good approximation of the rectus muscle is not necessary; the strength of the abdominal wall depends exclusively on the careful closure of the fascia.

(c) The anterior sheath of the rectus and fascia of the m. obliquus externus is closed by the overlapping method used in the radical cure of hernia.

(d) The skin is closed by a running stitch.

If exposure of the whole pelvis rather than of one side only is desired, the incision is made on the principles just described, but the oblique arms pass *to both* sides from the mid-line.

During the closure either of a uni- or bilateral incision, the insertion of a small drain for serum through the skin and sheath, down to the intermuscular space, proves a wise precaution.

The author insists on the unusually easy convalescence, as far as abdominal pains are concerned, of patients operated in this way; the slight amount of retraction needed may account for this good result.

